# Clinical dynamics of anhedonia symptom in mood disorder and in alcohol use disorder

**DOI:** 10.1192/j.eurpsy.2021.647

**Published:** 2021-08-13

**Authors:** O. Roshchina, G. Simutkin, N. Bokhan

**Affiliations:** 1 The Department Of Depressive States, Mental Health Research Institute, Tomsk National Research Medical Center of the Russian Academy of Sciences, Tomsk, Russian Federation; 2 Deaprtment Of Psychological Counseling And Psychotherapy, National Research Tomsk State University, Tomsk, Russian Federation

**Keywords:** anhedonia, mood disorders, alcohol use disorder

## Abstract

**Introduction:**

Anhedonia is an important transdiagnostic phenotypic characteristic of schizophrenia, mood disorders (MD), alcohol use disorder (AUD) and other mental diseases. This Symptom could reflect the neurochemical abnormalities in addictive and affective disorders when the function of reward system is dysregulated (Koob G.F., 2017).

**Objectives:**

To compare the severity of Anhedonia in clinic of MD and AUD in dynamic of antidepressant therapy

**Methods:**

The study enrolled 93 patients under treatment in MHRI Clinics: 45 AUD (F10.2; ICD-10) and 48 MD patients (F31-F34; ICD-10). The evaluation of Anhedonia was provided with the SHAPS modified for clinician administration (SHAPS-C) (Rezvan A., 2014).

**Results:**

Due to statistical analysis, we found the level of anhedonia in the MD group was higher than in the AUD group before the treatment. After four weeks of antidepressant therapy the scrutiny of score difference shows less changes in severity of the Symptom in the AUD group (Table 1) Table 1. Dynamics of Anhedonia in MD and AUD groups by SHAPS-C

**Table:** 

Group	Total score upon admission	Total score after four weeks therapy	Total score upon admission and after four weeks therapy
AUD (n=45)	24 (22; 27,5)	22 (20,25; 28)	1 (-3; 4)
MD (n=48)	30 (23; 38)	25 (20; 28)	4 (1; 8)
р (Mann-Whitney test)	0,003	0,373	0,002

**Conclusions:**

Anhedonia in the structure of AUD is less pronounced than in MD, but responds less to antidepressant therapy. The study is supported by RSF Grant no. 19-15-00023 “Clinical features and search of potential biomarkers of comorbidity of alcoholism and affective disorders”.

